# Validation and Improvement of a Rapid, CRISPR-Cas-Free RPA-PCRD Strip Assay for On-Site Genomic Surveillance and Quarantine of Wheat Blast

**DOI:** 10.3390/jof12010073

**Published:** 2026-01-18

**Authors:** Dipali Rani Gupta, Shamfin Hossain Kasfy, Julfikar Ali, Farin Tasnova Hia, M. Nazmul Hoque, Mahfuz Rahman, Tofazzal Islam

**Affiliations:** 1 Institute of Biotechnology and Genetic Engineering (IBGE), Gazipur Agricultural University, Gazipur 1706, Bangladesh; drgupta80@gmail.com (D.R.G.); shk4221agri@gmail.com (S.H.K.); julfikarhstu.ac.bd@gmail.com (J.A.); farintasnova1998@gmail.com (F.T.H.); 2 Molecular Biology and Bioinformatics Laboratory, Department of Gynaecology, Obstetrics and Reproductive Health, Gazipur Agricultural University, Gazipur 1706, Bangladesh; nazmul90@gau.edu.bd; 3 Extension Service, West Virginia University, Morgantown, WV 26506, USA; mm.rahman@mail.wvu.edu

**Keywords:** *Magnaporthe oryzae Triticum*, point-of-care detection, lateral flow assay, field diagnosis, early warning advisory system, IPM

## Abstract

As an emerging threat to global food security, wheat blast necessitates the development of a rapid and field-deployable detection system to facilitate early diagnosis, enable effective management, and prevent its further spread to new regions. In this study, we aimed to validate and improve a Recombinase Polymerase Amplification coupled with PCRD lateral flow detection (RPA-PCRD strip assay) kit for the rapid and specific identification of *Magnaporthe oryzae* pathotype *Triticum* (MoT) in field samples. The assay demonstrated exceptional sensitivity, detecting as low as 10 pg/µL of target DNA, and exhibited no cross-reactivity with *M. oryzae Oryzae* (MoO) isolates and other major fungal phytopathogens under the genera of *Fusarium*, *Bipolaris*, *Colletotrichum*, and *Botrydiplodia*. The method successfully detected MoT in wheat leaves as early as 4 days post-infection (DPI), and in infected spikes, seeds, and alternate hosts. Furthermore, by combining a simplified polyethylene glycol-NaOH method for extracting DNA from plant samples, the entire RPA-PCRD strip assay enabled the detection of MoT within 30 min with no specialized equipment and high technical skills at ambient temperature (37–39 °C). When applied to field samples, it successfully detected MoT in naturally infected diseased wheat plants from seven different fields in a wheat blast hotspot district, Meherpur, Bangladesh. Training 52 diverse stakeholders validated the kit’s field readiness, with 88% of trainees endorsing its user-friendly design. This method offers a practical, low-cost, and portable point-of-care diagnostic tool suitable for on-site genomic surveillance, integrated management, seed health testing, and quarantine screening of wheat blast in resource-limited settings. Furthermore, the RPA-PCRD platform serves as an early warning modular diagnostic template that can be readily adapted to detect a wide array of phytopathogens by integrating target-specific genomic primers.

## 1. Introduction

Wheat blast, caused by *Magnaporthe oryzae* pathotype *Triticum* (MoT), has emerged as one of the most destructive diseases threatening global wheat production and food security [1,2]. Since its first appearance in Brazil in 1985, the pathogen has spread across South America and was reported in Bangladesh in 2016, marking its first outbreak in Asia [3,4]. More recently, wheat blast occurrence in Zambia [2] has raised serious concerns about the transcontinental spread of this pathogen through seed and wind dispersal [5]. Considering that China and India, the world’s leading wheat producers, share borders with Bangladesh, the risk of disease incursion poses a significant food security threat [1].

This fungus predominantly attacks wheat spikes, leading to shriveled or deformed grains within days of infection and causing yield losses of up to 100% under favorable conditions [4]. The visual symptoms of wheat blast closely resemble those of Fusarium head blight, complicating accurate field diagnosis and management [6]. Despite decades of research, no fully resistant wheat cultivars are available, and current control relies largely on fungicide use and cultural practices, which provide limited and inconsistent protection. Because MoT can spread through infected seeds and crop residues, early and accurate detection of the pathogen is essential for effective quarantine enforcement, seed certification, genomic surveillance, and timely disease management [5]. Another complication is that MoT is predominantly a head disease and remains almost asymptomatic at the vegetative stage of wheat. As a result, once bleached head symptoms are seen in the field, fungicide application is ineffective in protecting against yield loss. Conventional diagnostic tools, such as PCR and qPCR, though reliable, require well-equipped laboratories, a stable power supply, and trained personnel, which limit their use in field settings [7,8]. In contrast, Recombinase Polymerase Amplification (RPA) is an innovative isothermal nucleic acid amplification method that enables rapid, sensitive, and instrument-free detection under constant low temperatures [9,10,11]. RPA reactions only require a pair of 30–35 bp primers and can be completed within 30 min at 37–42 °C [12,13]. The resulting amplicons can be analyzed using various methods, including gel electrophoresis, fluorescent probes, or lateral flow strips [14,15]. Among these, lateral flow strips provide a simple, rapid, and visual means of detecting amplification results with the naked eye [16,17]. Therefore, coupling RPA with lateral flow detection technology offers a powerful approach for visual, on-site identification of target pathogens within a short time.

Robust, concept-proven diagnostic technologies are crucial for mitigating plant diseases and minimizing yield losses through timely and accurate detection [17,18]. The recent development of CRISPR-Cas-based diagnostic assays for wheat blast represents a major breakthrough, as these tools have demonstrated high specificity and strong potential for precise pathogen identification [17,18,19,20,21]. This proof of concept underscores the potential of CRISPR-based technology to enhance genomic surveillance and containment of the wheat blast pathogen [22]. However, current CRISPR diagnostic methods often depend on temperature-sensitive reagents and multistep laboratory procedures, which limit their portability and field application [17,20]. Furthermore, there is an ongoing need to simplify DNA extraction and sample preparation to facilitate the on-site application of Recombinase Polymerase Amplification (RPA) in remote field settings [17].

Despite the urgent need for early and accurate detection, no large-scale, rapid, and field-deployable diagnostic kits are currently available for effective quarantine and genomic surveillance of wheat blast [22]. To address this gap, we developed and optimized an RPA-based molecular detection system coupled with lateral flow strip (PCRD) visualization. The assay was validated using infected leaves, spikes, and seeds and was further streamlined with a crude sap DNA extraction method based on alkaline polyethylene glycol (PEG-NaOH) to enable reliable on-site detection. This study aimed to (i) improve a rapid and convenient diagnostic kit for wheat blast by excluding the patented Cas12a enzyme and optimizing the DNA extraction process; (ii) evaluate and validate the efficacy of the kit using field samples, including wheat plants, spikes, seeds, and alternate grass hosts; (iii) validate diagnostic specificity against major fungal phytopathogens, including the rice blast fungus (*M. oryzae Oryzae*) and species from the genera *Fusarium*, *Bipolaris*, *Colletotrichum*, and *Botryodiplodia*; and (iv) train relevant stakeholders (researchers, extension workers, pathologists, and industry personnel) to assess the robustness, usability, and practical applicability of the developed kit.

## 2. Materials and Methods

### 2.1. Fungal Isolate Retrieval and Sub-Culture

Fungal isolates were retrieved from the culture repository of the Institute of Biotechnology and Genetic Engineering (IBGE), Gazipur Agricultural University (GAU), Bangladesh. *Colletotrichum gloeosporioides* and *Botryodiplodia theobromae* isolates were provided by Dr. Mynul Haque of Bangladesh Agricultural Research Institute, Bangladesh. Isolates of *Fusarium oxysporum*, *Magnaporthe oryzae Oryzae*, *Bipolaris sokiniana*, and other fungal isolates used were collected from the stored fungi of the Institute of Biotechnology and Genetic Engineering (IBGE) of Gazipur Agricultural University, Bangladesh. The isolates were sub-cultured onto 2% potato dextrose agar (PDA) medium in Petri dishes and incubated at 26 °C for 10 days. After incubation, the white mycelial mats were carefully scraped from the surface of the agar using a sterile spatula and collected for genomic DNA extraction.

### 2.2. Primers and Probe Used in RPA-PCRD Strip

The MoT-specific gene sequence MoT-6098 was selected as the target gene for designing the primers and probe for the RPA assay previously identified in [19]. Details of primers and probes used in the development of the RPA-PCRD kit are provided in Table 1. Forward primers were labeled with fluorescent 6-FAM (6-Carboxyfluorescein), while reverse primers were labeled with biotin. The probe has the following characteristics: 45 bp in length; 28 bases are on the 5′, followed by a tetrahydrofuran abasic site (THF) replacing a base between fluorophore and quencher; and finally, a C3 spacer block that prevents amplification. These primers for RPA used in the rapid diagnostic kit were designed by Peng Ye and her group (unpublished) of the Institute of Plant Protection of the Chinese Academy of Sciences, Beijing, China, and we received the kits from them.

### 2.3. DNA Extraction from Plant Sample and Fungal Isolates

Following a 5–10-day incubation period, depending on the growth of the fungi (Table 2) on PDA plates at 26 °C, the fungal mycelia were harvested by gently scraping them from the agar surface. Infected and healthy plant samples were collected for artificially inoculated plants or naturally infected fields. The collected mycelia and/or plant samples were ground thoroughly in a pre-chilled mortar and pestle to disrupt the cell walls. Genomic DNA was then extracted using the Wizard^®^ Genomic DNA Purification Kit (Promega Corporation, Madison, WI, USA) according to the manufacturer’s protocol. The purity and concentration of the extracted DNA were quantified using a fluorometer, and the DNA samples were diluted with sterile distilled water to the desired working concentrations for downstream applications.

### 2.4. Optimization of Recombinase Polymerase Amplification (RPA) Reaction

The commercial lateral flow (LF) strip, HybriDetect MGHD1—supplied by AMPfuture Biotech Ltd., Beijing, China—was applied to detect RPA amplicons visually. The LF strips were designed to detect amplicons labeled with biotin and 6-carboxy-fluorescein (FAM), which were obtained using an AMP-Future RPA kit (Biotech Co. Ltd.; Beijing, China). The RPA was conducted following the published method [23]. Each 50 µL reaction mixture was prepared in a microcentrifuge tube containing 29.4 µL of A buffer, 2 µL each of forward and reverse primers, 0.6 µL of nfo probe, 11.5 µL of ddH_2_O, and 2 µL of template genomic DNA (gDNA). The reaction was initiated by adding 2.5 µL of B buffer, and the mixture was immediately incubated at 39 °C for 10 min. To optimize reaction conditions, the reaction times (5, 8, 10, and 20 min) and temperatures (35–39 °C) were varied independently while keeping other parameters constant. PCRD strips were used to analyze the amplified products. A total of 10 μL of the amplicon was mixed with 80 μL of distilled water, and 70–75 μL of this mixture was applied onto the sample pad. Band development was monitored for up to 10 min, although results typically appeared within 2–3 min. The presence of control and test lines on the strip was visually inspected for qualitative assessment. The optimal conditions were determined based on the intensity of the amplified bands visualized on the PCRD strip.

### 2.5. Efficacy and Specificity Tests of RPA Assay

To determine the specificity of the RPA-PCRD strip assay, gDNA of five MoT isolates—five other pathogenic fungi strains—was subjected to RPA-PCRD strip assays (Table 1). To test the efficacy and specificity of the RPA assay, a 10-fold serial dilution of MoT gDNA was subjected to the RPA assay. The same amount of MoT gDNA was also subjected to a conventional PCR assay. The PCR reactions were prepared for a 10 μL volume containing 5 μL of DreamTaq Green PCR Master Mix (Thermo Scientific™, Waltham, MA, USA), 3 μL of nuclease-free water, 1 μL of 10 pmol/μL of each primer, and 1 μL of the template (the initial concentration of DNA was 50 ng/μL). PCR cycling conditions were 95 °C for 4 min, followed by 35 cycles of denaturation at 95 °C for 30 s, annealing at 60 °C for 30 s, extension at 72 °C for 40 s, and a final incubation at 72 °C for 10 min. The amplified products were visualized using the Molecular Imager R (GelDocTM XR, Bio-Rad Laboratories, Inc., Hercules, CA, USA). Both sensitivity and specificity tests were performed twice.

### 2.6. Preparation of Conidial Suspension and Artificial Inoculation of Wheat and Alternate Hosts

Conidia were induced and harvested from 10-day-old cultures of MoT grown on PDA plates, following the method described by Gupta, Surovy [24]. To collect the conidia, the culture plates were flooded with sterile distilled water, and the surface was gently brushed with a sterile paintbrush to dislodge the conidia. The resulting suspension was filtered through sterile cheesecloth to remove mycelial debris, and the conidial concentration was adjusted to 5 × 10^4^ conidia/mL using a hemocytometer.

Seeds of wheat (*Triticum aestivum* cv. BARI Gom 26), maize (*Zea mays*), barley (*Hordeum vulgare*), and oat (*Avena sativa*) were sown in plastic pots (12 cm × 7.5 cm) containing sterilized field soil, with 10–15 seeds per pot. Ten-day-old seedlings of wheat, barley, and oat, and fifteen-day-old maize seedlings, were sprayed with the conidial suspension of a virulent MoT isolate (BTJP 4-5) until run-off. Inoculated plants were maintained under 16 h of light per day, with 80–90% relative humidity at 26 °C and grown under natural sunlight conditions.

### 2.7. RPA-PCRD Strip Assay Using Wheat Blast-Infected Field Samples

Field samples of wheat blast were collected during the 2023–2024 growing season from multiple sites in the wheat blast hotspot district, Meherpur, Bangladesh [4]. Samples were taken from different plant parts such as leaves, spikes, and seeds to ensure diverse representation of infection stages and symptom types. Additionally, seeds were collected from a controlled, artificially inoculated wheat plot that exhibited 20.22% disease incidence and 4.09% disease severity, providing well-characterized samples for comparison and validation of the PCR and RPA-PCRD strip assays. All collected samples were stored at –80 °C until further processing.

For field validation of the RPA-PCRD strip assay, a modified polyethylene glycol (PEG)–NaOH method was used to extract crude DNA from infected plant samples (detailed procedures are described in Section 3). A pre-mixed cocktail containing all reagents, primers, and the probe was used to minimize the risk of contamination, as developed by the Institute of Plant Protection of the Chinese Academy of Agricultural Sciences, Beijing, China. The freeze-dried enzyme pellet was reconstituted in 49 µL of nuclease-free water, and 2 µL of crude DNA extract was added. The reaction mixture was incubated using body heat at ambient temperature (37–39 °C) for 10 min. The resulting product was then diluted as described earlier and applied to PCRD lateral flow strips for visualization of results.

## 3. Results

### 3.1. Efficacy Assessment of an RPA-PCRD Assay for Wheat Blast Detection

**Figure 1:**
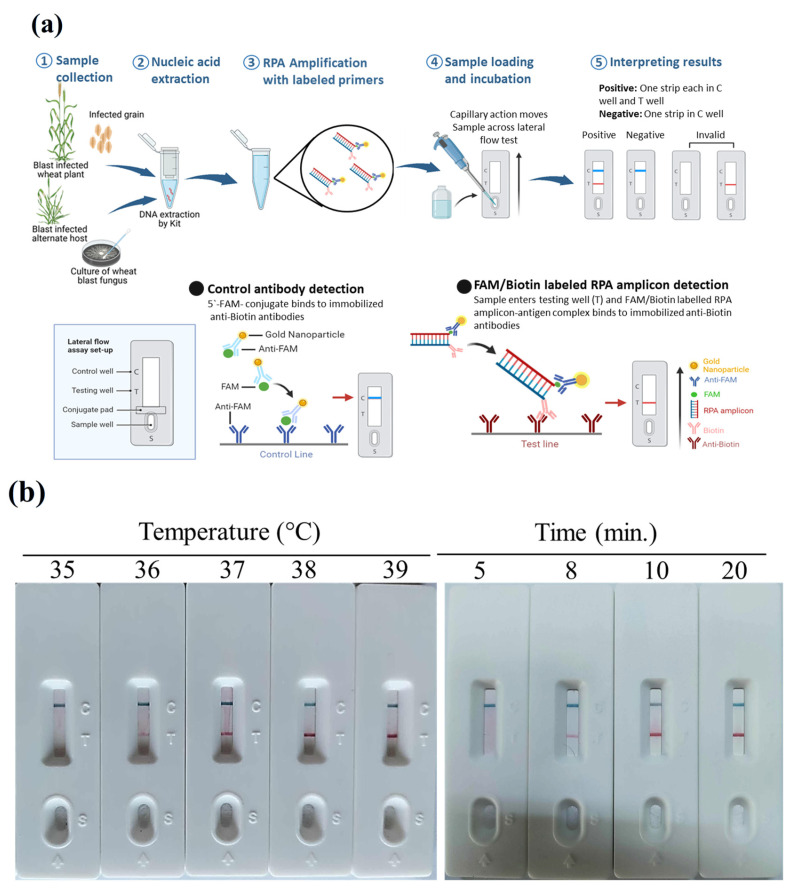

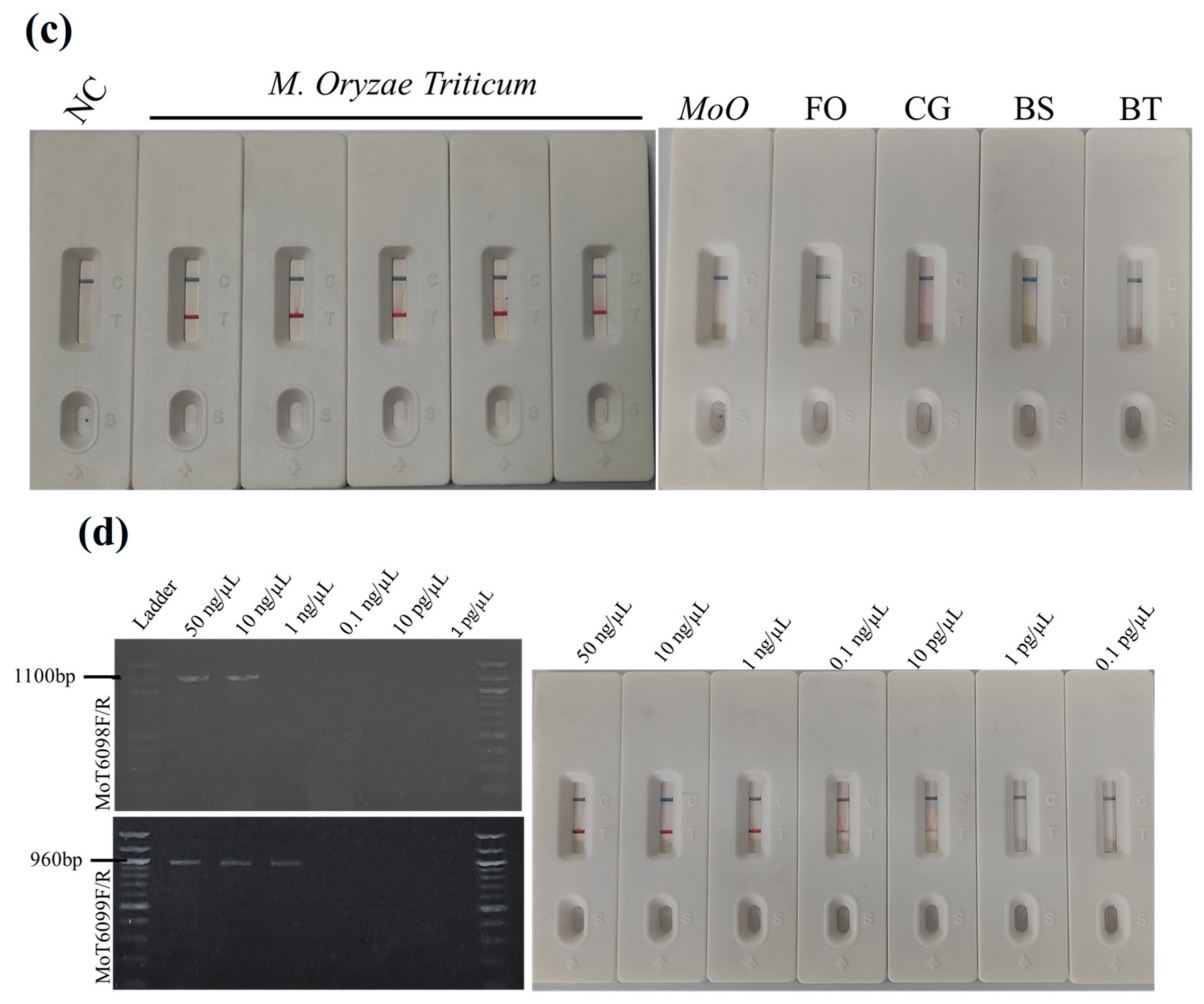
RPA-PCRD strip-based assay for wheat blast detection. (**a**) Schematic diagram of RPA-PCRD strip-based detection of MoT fungus from various samples in the laboratory. (**b**) Determination of optimum temperature and time for successful detection of the MoT fungus using the developed RPA-PCRD strip assay. The primer/probe sets are labeled with Biotin/FAM. RPA reactions were carried out in an Eppendorf tube at various temperatures and time intervals, and samples were loaded onto the PCRD strips. (**c**) Specificity of RPA-PCRD strip assay for MoT detection. DNA was extracted from the fungal isolates, RPA reaction was carried out at 39 °C for 10 min, and samples were loaded onto PCRD strips (NC: negative; MoO: *M. oryzae Oryzae*; FS: *Fusarium oxysporum*; CG: *Colletotrichum gloeosporioides*; BP: *Bipolaris sorokiana*; BT: *Botryodiplodia theobromae*). (**d**) Sensitivity assay of RPA-PCRD strip assay. A 10-fold genomic DNA serial dilution (50 ng–0.01 pg) was used to perform the sensitivity assays MoT-specific primers using MoT6098F/R, MoT6099F/R (**left** panel), and RPA-PCRD strip (**right** panel). Each experiment was repeated three times, and each treatment was replicated at least five times (n = 5) to ensure reproducibility.

To circumvent the temperature constraints of the Cas12a enzyme and navigate intellectual property restrictions, we improved a CRISPR-free, isothermal detection platform utilizing an RPA-PCRD (Recombinase Polymerase Amplification—Polymerase Chain Reaction Detectable) strip for field validation. The efficacy of the system was evaluated using *Magnaporthe oryzae Triticum* (MoT) genomic DNA, following the workflow illustrated in Figure 1a.

The RPA reaction was first optimized for temperature and incubation time. Under isothermal conditions across a gradient of 35–39 °C, distinct test bands were visible on the lateral flow strips from 36 °C to 39 °C (Figure 1b). Maximum signal intensity (the most robust bands) was observed between 37 °C and 39 °C; consequently, 39 °C was established as the standard temperature for subsequent assays. Temporal optimization showed that while positive amplification was detectable as early as 8 min, the signal was relatively weak. Incubation periods of 10–20 min yielded optimal, high-intensity bands. Therefore, a 10 min reaction time was selected to ensure rapid turnaround without compromising sensitivity.

The specificity of the RPA-PCRD assay was challenged using five *MoT* isolates, five *MoO* (rice blast) isolates, and five diverse fungal species. The assay successfully amplified all five *MoT* isolates, while no amplification was observed for *MoO* or any other fungal species (Figure 1c). This absolute specificity ensures the accurate differentiation of wheat blast from other *Magnaporthe* pathotypes common in rice-growing regions. Furthermore, the assay showed no cross-reactivity with other major wheat head-infecting pathogens, such as *Fusarium* and *Bipolaris*, even at high DNA concentrations.

Serial dilutions of *MoT* genomic DNA (1 to 0.0001 ng/µL) were used to benchmark the analytical sensitivity of the RPA-PCRD assay against conventional PCR. Using primer sets *MoT6098F/R* and *MoT6099F/R*, conventional PCR limits of detection (LODs) were 10 ng/µL and 1 ng/µL, respectively. In contrast, the RPA-PCRD assay achieved an LOD of 10 pg/µL (Figure 1d). This represents a 1000-fold increase in sensitivity over conventional PCR, demonstrating the platform’s potential for identifying low-titer infections in field-collected samples.

### 3.2. Validation of RPA-PCRD Using Field Samples and Alternate Hosts

**Figure 2:**
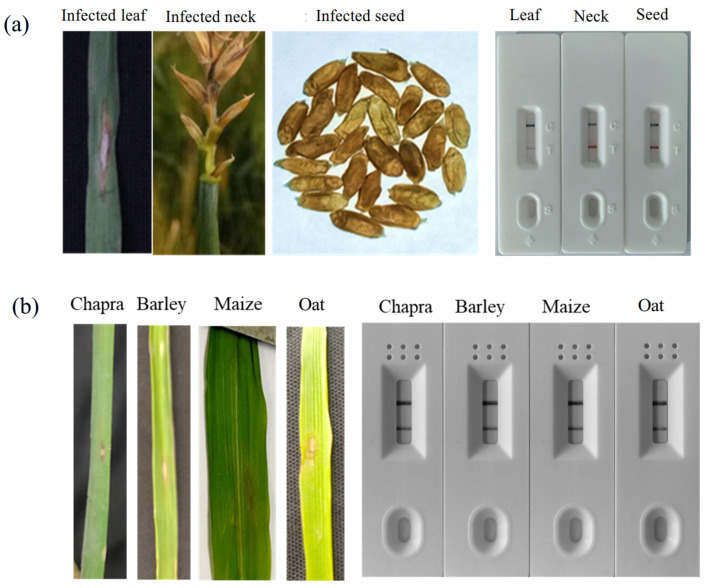
On-site application of RPA-PCRD strip for detection of wheat blast in host and alternate host. blast infected wheat samples. (**a**) RPA-PCRD strip-based assay for the detection of MoT in field samples and (**b**) alternate host. Test for each test sample was repeated three times.

To evaluate the diagnostic utility of the RPA-PCRD assay for field applications, we tested naturally infected wheat tissues (leaves, spike necks, and seeds) alongside various alternate host species. The assay consistently detected *MoT* DNA across all infected wheat tissues, confirming the presence of the pathogen in multiple plant matrices. In contrast, no amplification signals were observed in samples from healthy wheat plants, reinforcing the high specificity of the method (Figure 2a).

The robustness of the RPA-PCRD assay was further validated by its ability to detect *MoT* in artificially inoculated alternate hosts, including oat, maize, and barley (Figure 2b). Given that these species often serve as environmental reservoirs for the fungus [25], the assay’s ability to identify the pathogen across diverse cereal hosts highlights its versatility for broader epidemiological monitoring.

In addition to inoculated samples, we screened several weeds and asymptomatic grasses collected from the periphery of infected wheat fields to investigate potential natural reservoirs. However, none of these environmental samples yielded a positive reaction (Figure S1). These findings collectively demonstrate that the RPA-PCRD assay effectively distinguishes *MoT*-infected materials from healthy plant tissues and can reliably identify the pathogen across a range of susceptible hosts, facilitating rapid and accurate on-site surveillance.

### 3.3. Early Detection of MoT in Wheat Leaves

Since wheat blast is often asymptomatic during the vegetative stage, detecting the pathogen prior to the heading stage is critical; the disease spreads rapidly via conidia and can cause catastrophic yield losses under favorable environmental conditions. Early identification allows for the timely implementation of control measures, preventing regional outbreaks and safeguarding production. To evaluate the early diagnostic potential of the RPA-PCRD assay, wheat seedlings were inoculated with *MoT* conidia and sampled at specific intervals from 3 to 10 days post-inoculation (DPI). Visible symptoms characterized by small, water-soaked lesions first appeared at 6 DPI. DNA was extracted from both symptomatic and asymptomatic leaf tissues for comparative analysis.

**Figure 3:**
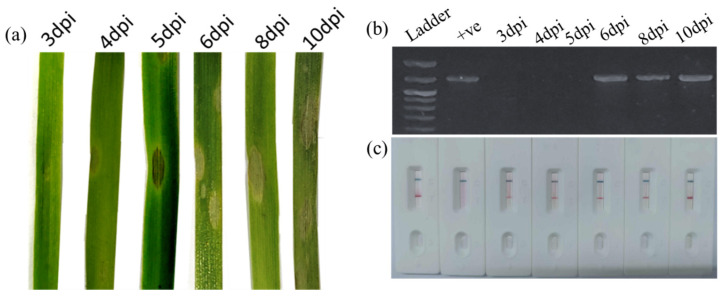
Early detection of MoT in artificially inoculated wheat leaves. (**a**) Progression of visual symptoms in wheat leaves at various days post-inoculation (DPI) with MoT. (**b**) Conventional PCR and (**c**) RPA-PCRD strip assay for the detection of MoT in wheat leaf samples collected at various days post-inoculation (DPI) with MoT. Tests for each test sample were repeated three times.

While conventional PCR was only able to detect *MoT* starting at 6 DPI (coinciding with symptom onset), the RPA-PCRD assay successfully detected the pathogen as early as 4 DPI. This represents a two-day lead time before symptoms become visible (Figure 3). These results demonstrate the superior analytical sensitivity of the RPA-PCRD method, establishing its efficacy for pre-symptomatic diagnosis and enabling proactive disease management interventions to arrest further spread.

### 3.4. Optimization of DNA Extraction

**Figure 4:**
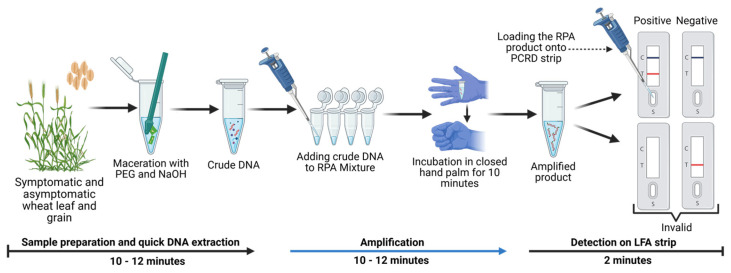
Schematic steps involve performing RPA-PCRD strip assay for on-field detection of wheat blast fungus. Step 1: Plant samples were processed using PEG-NaOH buffer and a micro pestle. Step 2: The crude extract was directly used in RPA reactions as a template and incubated using closed hand palm for 10 min. Step 3: Visual detection of RPA results on RPA-PCRD strips. All the reactions were carried out using a cocktail of TwistAmp-nfo kit provided by the Institute of Plant Protection of the Chinese Academy of Agricultural Sciences, Beijing, China. Total procedure completed within 30 min.

To facilitate the deployment of the RPA-PCRD assay in field or low-resource settings, we optimized a rapid polyethylene glycol (PEG)–NaOH lysis method for total DNA extraction. This alkaline-based protocol yields crude DNA in approximately 10–15 min and proved effective across diverse infected matrices, including leaf, neck, and seed tissues. In this study, infected leaf sections (5 × 5 mm), neck tissues (0.5 cm), or pooled seed samples (three seeds per sample) were homogenized in 1.5 mL microcentrifuge tubes. The homogenized material was lysed in 300 mL of 6% PEG200 containing 1 M NaOH. Following 1–2 min of manual agitation at room temperature, the lysate was left to stand for 10 min. Subsequently, 5 mL of the resulting supernatant was added directly to 45 mL of the RPA reaction mixture. After a 10 min incubation, the reaction was diluted with 50 mL of distilled water, and 70–80 mL of the final solution was loaded onto the PCRD strip. The entire diagnostic workflow, from sample preparation to visual result interpretation, was completed within 30 min without the requirement for specialized laboratory equipment or thermal cycling. The optimized procedural framework for the RPA-PCRD strip assay is illustrated in Figure 4.

### 3.5. Validation of RPA-PCRD Assay Using Field-Infected Samples

In March 2024, 50 field samples comprising 10 symptomatic leaves, 10 infected wheat necks, and 30 symptomatic or suspected seeds were collected from seven distinct fields in Meherpur, Bangladesh. While the leaf and neck samples exhibited clear clinical symptoms of wheat blast, the seed samples displayed varying degrees of suspected infection without uniform visible signs. To compare diagnostic efficiency, each leaf and neck sample was partitioned into two equivalent portions: one for high-purity DNA extraction (commercial kit) followed by conventional PCR and the other for rapid PEG-NaOH-based extraction followed by the RPA-PCRD strip assay. For seed analysis, five seeds were randomly selected from each infected spike, homogenized, and divided for parallel testing using the same two methodologies.

**Figure 5:**
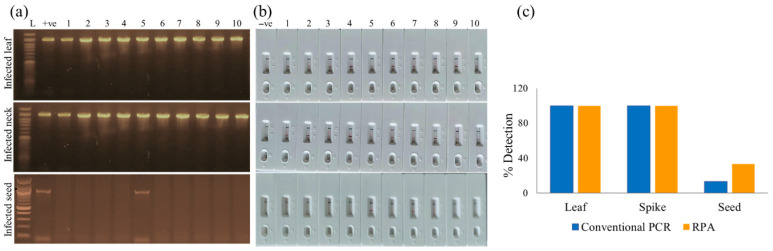
Validation of the RPA-PCRD assay for the detection of MoT in wheat blast in infected leaf, spike, and seed samples (n = 5) collected from Meherpur (2023–2024). Suspected infected parts of the leaf and rachis tissues of the spikes and single seeds were used in extraction of DNA. (**a**) Conventional PCR was performed using MoT-6098F/R primers and DNA extracted by a kit, (**b**) RPA-PCRD strip assay using NaOH-PEG extracted crude DNA, and (**c**) bar graph showing the comparison of % detection of field sample between conventional PCR and RPA-PCRD strip assay. We repeated each experiment thrice to ensure reproducibility. +ve, positive for MoT, and -ve, negative for MoT; L, ladder.

The RPA-PCRD assay demonstrated 100% diagnostic agreement with conventional PCR for all vegetative tissues; all 10 leaf and 10 neck samples yielded distinct positive test lines on the strips and corresponding bands in the PCR assays (Figure 5a,b). Notably, the RPA-PCRD system exhibited superior sensitivity in seed samples. While conventional PCR detected the pathogen in only one seed sample (Figure 5b, lower panel), the RPA-PCRD assay identified two positive samples, doubling the detection rate of the traditional method (Figure 5c). In contrast, all healthy control samples collected from the same sites tested negative, confirming the assay’s high specificity. These findings validate the RPA-PCRD system as a rapid, sensitive, and reliable tool for the practical field detection of *Magnaporthe oryzae* Triticum (MoT).

### 3.6. Train Stakeholders to Assess the Robustness, Usability, and Practical Applicability of the Developed Kit

To train relevant stakeholders (researchers, extension workers, pathologists, and industry personnel), we arranged a day-long workshop to discuss the concept and then demonstrate the specificity and convenience of the developed wheat blast diagnostic kit to 52 trainees. Then, the trainees used this method using extracted DNA samples from MoT, MoO, *Fusarium* sp., and a control (no DNA). In both the demonstration and hands-on training session, the accuracy of the RPA-PCRD strips was recorded—98% (n = 250). More than 88% of the trainees opined that the kit is user-friendly.

## 4. Discussion

The escalating globalization of agricultural trade significantly heightens the risk of inadvertently spreading invasive phytopathogens, such as *Magnaporthe oryzae Triticum* (MoT), across international borders [1,26]. The 2016 introduction of MoT to Bangladesh, which devastated 1500 hectares of wheat within the first year, serves as a stark reminder of the vulnerability of regional food systems to seed-borne fungal pathogens [4]. While previous efforts utilized CRISPR-Cas12-based platforms for MoT detection, their reliance on temperature-sensitive enzymes and complex multistep protocols limits their utility in resource-constrained field settings [17]. In this study, we developed a streamlined and highly sensitive RPA–PCRD-based method for the rapid diagnosis of wheat blast fungus, MoT in seeds, infected plants, and alternative hosts. While our approach builds upon previously reported CRISPR-Cas12-based platforms, it overcomes significant barriers to practical field deployment. Although CRISPR-Cas12 systems demonstrate high sensitivity, their utility in remote settings is often constrained by the temperature sensitivity of Cas enzymes and the operational complexity of multistep protocols [17].

The hallmark of our refined method is its superior efficiency and operational simplicity; while the system reported by Kang, Peng [19] requires two rounds of RPA followed by a 25–30 min CRISPR-Cas12 digestion step, our assay utilizes a single RPA reaction completed within just 10 min. This optimization significantly reduces the total diagnostic turnaround time and eliminates the need for specialized CRISPR-associated reagents and complex biochemical handling. Furthermore, unlike many molecular diagnostics that rely on sophisticated laboratory infrastructure and highly trained personnel, our RPA-based technique is robust and user-friendly. Operating efficiently at a constant temperature of 37–39 °C, the assay possesses the thermal flexibility to be powered by basic heat sources—such as portable incubators, water baths, or even ambient human body heat. These features make the method exceptionally well-suited for on-site surveillance and quarantine screening in resource-limited environments.

In this study, we targeted the MoT-6098 gene, which encodes a unique acid trehalase protein to ensure absolute diagnostic specificity for the wheat blast fungus (*Magnaporthe oryzae Triticum*, or MoT) [19]. The specificity of our RPA-PCRD system was confirmed by the absence of detection signals when tested against the rice blast fungus (MoO), other wheat pathogens such as *Fusarium* and *Bipolaris*, and several major phytopathogenic fungi. Furthermore, the system showed no cross-reactivity with healthy wheat tissue or background microbial DNA, reinforcing its reliability. By integrating a PEG-NaOH-based extraction method, we achieved rapid, high-quality DNA recovery suitable for field conditions, marking a significant advancement in the point-of-care detection of wheat blast.

To maximize diagnostic performance, we optimized the RPA reaction kinetics, specifically evaluating the impact of incubation time and temperature on signal intensity. While the PCRD strip produced a detectable signal in as little as 8 min, the band intensity, which correlates directly with the concentration of the amplified target, was significantly more robust at 10 min. Consequently, a 10 min incubation was selected to ensure an optimal balance between rapid turnaround and high sensitivity (Figure 1b).

Our RPA-PCRD assay achieved a Limit of Detection (LOD) of 10 pg/µL, completed within a total timeframe of 30 min. This level of sensitivity is consistent with previous findings where RPA-based detection surpassed conventional PCR by 100-fold [27] and exceeded LAMP-based methods by 10-fold [28]. Crucially, in field-collected samples, the RPA-PCRD strip outperformed conventional PCR in MoT detection rates. While detection was more consistent in leaf and neck tissues than in seeds, this discrepancy is rooted in the pathology of MoT. MoT primarily colonizes the wheat neck, which disrupts nutrient translocation; this often results in shriveled, symptomatic seeds that may not harbor high titers of the pathogen itself [29]. The practical utility of this method is further enhanced by its compatibility with crude plant sap. By bypassing labor-intensive DNA extraction, the total diagnostic window is reduced to approximately 20 min. This combination of high analytical sensitivity, biological reliability across different tissue types, and field-ready simplicity makes the RPA-PCRD system an ideal tool for real-time disease surveillance and high-stakes quarantine screening at ports and borders.

**Figure 6:**
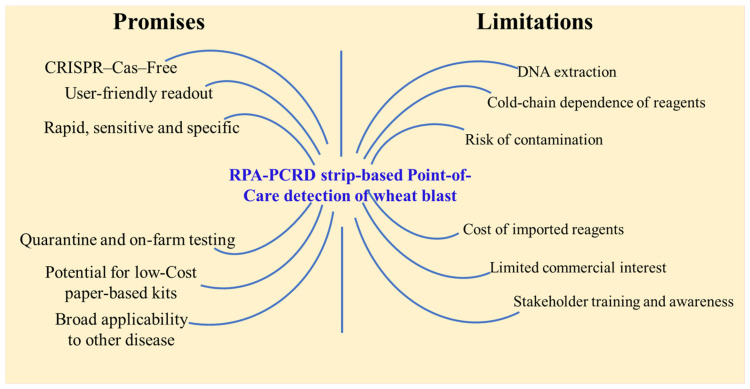
Promises and limitations of the newly developed RPA-PCRD strip assay for the detection of wheat blast in farmers’ fields.

The primary advantage of the validated RPA-PCRD method lies in its operational simplicity and superior sensitivity compared to conventional PCR and CRISPR-Cas-based systems. Our assay operates under mild isothermal conditions (37–39 °C) and requires minimal equipment, contrasting sharply with the thermal cycling and specialized laboratory infrastructure necessitated by traditional methods. While the detection limit for conventional PCR in MoT diagnosis is approximately 0.1 ng/μL [19], our RPA-PCRD assay achieved a significantly lower detection limit of 10 pg/μL. Although quantitative real-time PCR (qPCR) can reach comparable sensitivity (10 pg/μL), it remains constrained by its requirement for sophisticated instrumentation and precise thermal protocols. In contrast, the RPA-PCRD strip format delivers high-sensitivity results within 30 min using only basic heating equipment (Figure 6). Furthermore, the robustness of our assay eliminates the need for high-purity DNA templates, which are mandatory for both conventional PCR and other previously described molecular methods. By utilizing crude templates, we significantly reduce both the total turnaround time and the per-sample cost. While a formal economic analysis was not performed, the absence of specialized laboratory equipment and additional reagents suggests that our diagnostic kit is a cost-effective solution; however, a comparative study with traditional PCR remains essential for assessing its commercial viability. These attributes—high analytical sensitivity, tolerance to inhibitors, and rapid visual interpretation—make the RPA-PCRD system exceptionally well-suited for on-site agricultural inspections, port-of-entry quarantine screening, and field-based monitoring programs.

The newly developed CRISPR-Cas-free RPA-PCRD strip assay holds significant promise for the real-time surveillance of wheat blast, particularly in resource-limited environments (Figure 6). Its primary advantages are its simplicity and rapid turnaround, delivering highly sensitive, specific, and visually interpretable results within 30 min at a constant isothermal temperature (39–42 °C). This eliminates the requirement for sophisticated thermal cyclers, making the assay inherently field-deployable. The high analytical sensitivity of the platform enables the detection of low-titer pathogen loads, which is critical for identifying infections during asymptomatic or early colonization phases. By bypassing the technical complexities and licensing constraints inherent to CRISPR-Cas systems, this assay offers a more cost-effective and accessible “point-of-care” solution for farmers and plant health officials [18]). Furthermore, because the method operates independently of specialized laboratory infrastructure, it is uniquely suited for decentralized applications, such as on-farm genomic surveillance and remote field monitoring. Given that wheat is a cornerstone of global food security, this tool has immediate utility at strategic biosecurity nodes, including quarantine checkpoints, seed inspection units, and international border facilities. The transition toward this paper-based diagnostic format also offers a viable pathway to minimize production costs and enhance large-scale accessibility in developing regions. Beyond its current application for *M. oryzae Triticum*, the inherent modularity of this RPA-based platform allows for rapid adaptation to other emerging phytopathogens by simply substituting target-specific primers and probes.

Despite its significant advantages, the commercialization of this assay faces several challenges (Figure 6). While the PCRD strip simplifies visualization, the preliminary DNA extraction step currently necessitates basic laboratory equipment to ensure high-quality templates from complex plant tissues. Additionally, although the assay is robust, the potential for aerosol contamination remains a concern in field environments if reaction tubes are opened post-amplification. Future refinements focusing on a fully closed-tube system or integrated “crude” DNA extraction protocols will be essential to maximize the assay’s utility for direct on-site application by non-specialists. Furthermore, the localized nature of wheat blast outbreaks may currently limit immediate commercial interest in certain regions [17]. While the platform is field-ready, its large-scale deployment requires further simplification of the extraction workflow and rigorous validation under diverse “real-world” field conditions. Given that a majority of trainees (88%) perceived the kit as user-friendly, successful adoption will depend on comprehensive training programs to build technical confidence among end-users, including farmers, quarantine officers, and biosecurity policymakers. Logistically, the requirement for cold-chain storage and the importation of specialized RPA reagents may increase operational costs and lead to supply chain delays in remote areas [17]. Moreover, the high analytical sensitivity of the RPA-PCRD system necessitates strict adherence to standardized handling procedures to mitigate the risk of false-positive results. Nevertheless, this validated and improved diagnostic kit represents a critical advancement in wheat blast management. By enabling real-time genomic surveillance and rapid on-site detection, the assay provides a robust tool to enhance field management through an early warning advisory system and prevent the transboundary spread of the pathogen through international grain trade. Finally, the RPA-PCRD platform serves as a modular diagnostic template that can be readily adapted to detect a wide array of other phytopathogens by integrating target-specific genomic primers.

## Figures and Tables

**Table 1:** Primers used in the development of RPA-PCRD strip.

Primer	Sequence	Reference/Use
MoT6098 F	ACCAATATCACCTGAACGCAGACAT	Conventional PCR
MoT6098 R	GATTCCAGATTCACCACCAAAACAG	[19]
P98-1dRP 3F	TAACGGGCAGTCGCTAATGGTGTAGGTACTT	Conventional PCR
P98-1dRP3R	CTTGATTCTCTTGGGCTCCTGGCATTTCGG	Conventional PCR
F-P981dRP-3F	5′-[FAMdT]TAACGGGCAGTCGCTAATGGTGTAGGTACTT	For RPA by Peng Ye and her group (unpublished)
B-P981dRP-3R	5′-BiotinCTTGATTCTCTTGGGCTCCTGGCATTTCGG	For RPA by Peng Ye and her group (unpublished)
NfoPC-P981dRP	GCCTCACTTTACCGATTTGCTGGTCGAA (THF)CATGTGGCAGTGTCCTC, 3′, C3Spacer	For RPA by Peng Ye and her group (unpublished)

**Table 2:** Fungal isolates used in this study.

Pathogen	Isolate	Host Plant
*Magnaporthe oryzae Triticum*	BTJP 3-1	Wheat
*M. oryzae Triticum*	BTJP 4-1	Wheat
*M. oryzae Triticum*	BTJP 4-5	Wheat
*M. oryzae Triticum*	BTMP 1845-3	Wheat
*M. oryzae Triticum*	BTMP 1839-2	Wheat
*M. oryzae Oryzae*	RB13b	Rice
*M. oryzae Oryzae*	RbMe1819-3	Rice
*M. oryzae Oryzae*	RBTa 1849-2	Rice
*M. oryzae Oryzae*	RbMe 1816-2	Rice
*M. oryzae Oryzae*	Br48	Rice
*Bipolaris sorokiniana*	IBGEBs-2402	Wheat
*Fusarium oxysporum*	BTFD1	Dragon fruit
*Colletotrichum gloeosporioides*	MHPR2	Mango
*Botryodiplodia theobromae*	MAHR5	Mango

## Data Availability

The original contributions presented in this study are included in the article/Supplementary Material. Further inquiries can be directed to the corresponding author.

## Supplementary Materials

The following supporting information can be downloaded at https://www.mdpi.com/article/10.3390/jof12010073/s1: Figure S1: Application of RPA-PCRD-strip for detection of wheat blast in alternate host/weed. weed samples were collected from blast infected wheat field in Meherpur and subjected to RPA-PCRD strip-based assay for the detection of MoT. (a) photographs of symptomatic weed samples (b) RPA-PCRD strip-based assay for the detection of MoT in weed samples.

## References

[1] Islam M.T., Gupta D.R., Hossain A., Roy K.K., He X., Kabir M.R., Singh P.K., Khan M.A.R., Rahman M., Wang G.-L. (2020). Wheat blast: A new threat to food security. Phytopathol. Res..

[2] Tembo B., Mulenga R.M., Sichilima S., M’siska K.K., Mwale M., Chikoti P.C., Singh P.K., He X., Pedley K.F., Peterson G.L. (2020). Detection and characterization of fungus (Magnaporthe oryzae pathotype Triticum) causing wheat blast disease on rain-fed grown wheat (*Triticum aestivum* L.) in Zambia. PLoS ONE.

[3] Igarashi S. (1986). Pyricularia em trigo. 1. Ocorrencia de Pyricularia sp noestado do Parana. Fitopatol. Bras..

[4] Islam M.T., Croll D., Gladieux P., Soanes D.M., Persoons A., Bhattacharjee P., Hossain M.S., Gupta D.R., Rahman M.M., Mahboob M.G. (2016). Emergence of wheat blast in Bangladesh was caused by a South American lineage of Magnaporthe oryzae. BMC Biol..

[5] Latorre S.M., Were V.M., Foster A.J., Langner T., Malmgren A., Harant A., Asuke S., Reyes-Avila S., Gupta D.R., Jensen C. (2023). Genomic surveillance uncovers a pandemic clonal lineage of the wheat blast fungus. PLoS Biol..

[6] Ha X., Koopmann B., von Tiedemann A. (2016). Wheat blast and Fusarium head blight display contrasting interaction patterns on ears of wheat genotypes differing in resistance. Phytopathology.

[7] Bhat A.I., Aman R., Mahfouz M. (2022). Onsite detection of plant viruses using isothermal amplification assays. Plant Biotechnol. J..

[8] Venbrux M., Crauwels S., Rediers H. (2023). Current and emerging trends in techniques for plant pathogen detection. Front. Plant Sci..

[9] Zaghloul H., El-Shahat M. (2014). Recombinase polymerase amplification as a promising tool in hepatitis C virus diagnosis. World J. Hepatol..

[10] Daher R.K., Stewart G., Boissinot M., Bergeron M.G. (2016). Recombinase polymerase amplification for diagnostic applications. Clin. Chem..

[11] Babu B., Ochoa-Corona F.M., Paret M.L. (2018). Recombinase polymerase amplification applied to plant virus detection and potential implications. Anal. Biochem..

[12] Vasileva Wand N.I., Bonney L.C., Watson R.J., Graham V., Hewson R. (2018). Point-of-care diagnostic assay for the detection of Zika virus using the recombinase polymerase amplification method. J. Gen. Virol..

[13] Li C., Ju Y., Shen P., Wu X., Cao L., Zhou B., Yan X., Pan Y. (2021). Development of recombinase polymerase amplification combined with lateral flow detection assay for rapid and visual detection of Ralstonia solanacearum in tobacco. Plant Dis..

[14] Xu C., Li L., Jin W., Wan Y. (2014). Recombinase polymerase amplification (RPA) of CaMV-35S promoter and nos terminator for rapid detection of genetically modified crops. Int. J. Mol. Sci..

[15] Hu S., Yan C., Yu H., Zhang Y., Zhang C.-Q. (2023). Establishment of the recombinase polymerase amplification–lateral flow dipstick detection technique for Fusarium oxysporum. Plant Dis..

[16] Posthuma-Trumpie G.A., Korf J., van Amerongen A. (2009). Lateral flow (immuno) assay: Its strengths, weaknesses, opportunities and threats. A literature survey. Anal. Bioanal. Chem..

[17] Kasfy S.H., Hia F.T., Islam T. (2024). Do CRISPR-based disease diagnosis methods qualify as point-of-care diagnostics for plant diseases?. Nucleus.

[18] Islam T., Kasfy S.H. (2023). CRISPR-based point-of-care plant disease diagnostics. Trends Biotechnol..

[19] Kang H., Peng Y., Hua K., Deng Y., Bellizzi M., Gupta D.R., Mahmud N.U., Urashima A.S., Paul S.K., Peterson G. (2021). Rapid detection of wheat blast pathogen Magnaporthe oryzae Triticum pathotype using genome-specific primers and Cas12a-mediated technology. Engineering.

[20] Sánchez E., Ali Z., Islam T., Mahfouz M. (2022). A CRISPR-based lateral flow assay for plant genotyping and pathogen diagnostics. Plant Biotechnol. J..

[21] Tanny T., Sallam M., Soda N., Nguyen N.-T., Alam M., Shiddiky M.J. (2023). CRISPR/Cas-based diagnostics in agricultural applications. J. Agric. Food Chem..

[22] Islam T. (2024). Genomic surveillance for tackling emerging plant diseases, with special reference to wheat blast. CABI Rev..

[23] Lu X., Zheng Y., Zhang F., Yu J., Dai T., Wang R., Tian Y., Xu H., Shen D., Dou D. (2020). A rapid, equipment-free method for detecting *Phytophthora infestans* in the field using a lateral flow strip-based recombinase polymerase amplification assay. Plant Dis..

[24] Gupta D.R., Surovy M.Z., Mahmud N.U., Chakraborty M., Paul S.K., Hossain M.S., Bhattacharjee P., Mehebub M.S., Rani K., Yeasmin R. (2020). Suitable methods for isolation, culture, storage and identification of wheat blast fungus Magnaporthe oryzae Triticum pathotype. Phytopathol. Res..

[25] Durante L.G.Y., Bacchi L.M.A., Souza J.E.d., Graichen F.A.S. (2018). Reaction of wheat plants and alternative hosts to Magnaporthe oryzae. Arq. Inst. Biológico.

[26] Hulme P.E. (2021). Unwelcome exchange: International trade as a direct and indirect driver of biological invasions worldwide. One Earth.

[27] Wang Y., Zhang W., Wang Y., Zheng X. (2006). Rapid and sensitive detection of Phytophthora sojae in soil and infected soybeans by species-specific polymerase chain reaction assays. Phytopathology.

[28] Dai T., Yang X., Hu T., Jiao B., Xu Y., Zheng X., Shen D. (2019). Comparative evaluation of a novel recombinase polymerase amplification-lateral flow dipstick (RPA-LFD) assay, LAMP, conventional PCR, and leaf-disc baiting methods for detection of Phytophthora sojae. Front. Microbiol..

[29] Valent B., Cruppe G., Stack J.P., Cruz C., Farman M.L., Paul P.A., Peterson G.L., Pedley K.F. (2021). Recovery plan for wheat blast caused by Magnaporthe oryzae pathotype Triticum. Plant Health Prog..

