# Supplementary material for: Validation and Improvement of a Rapid, CRISPR-Cas-Free RPA-PCRD Strip Assay for On-Site Genomic Surveillance and Quarantine of Wheat Blast
Source: J Fungi (Basel). 2026 Jan 18;12(1):73. doi: 10.3390/jof12010073 (PMC12842714; doi:10.3390/jof12010073)
Supplement: Supplementary file 1 [file jof-12-00073-s001.zip › jof-4101236-supplementary.pdf]

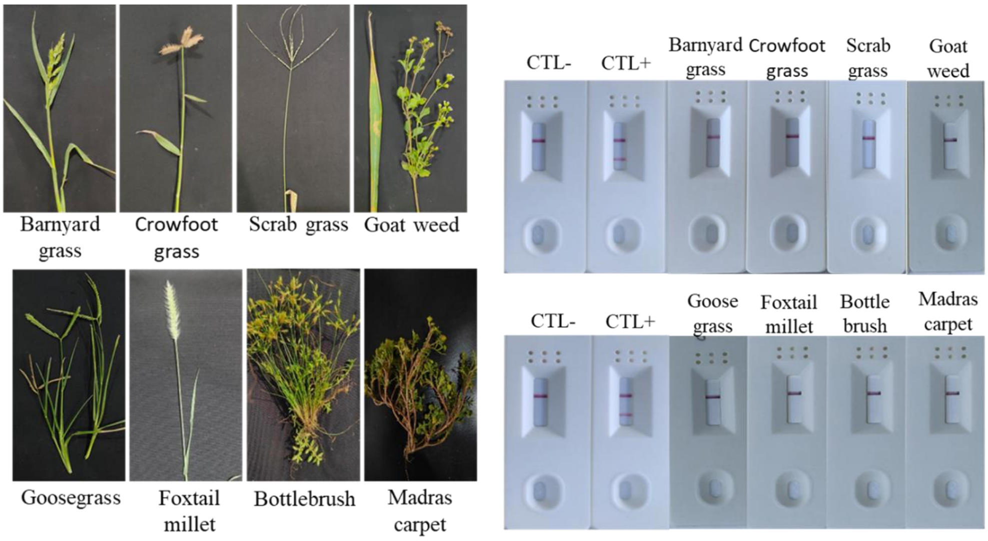


**Figure S1.** Application of RPA-PCRD-strip for detection of wheat blast in alternate host/weed. weed samples were collected from blast infected wheat field in Meherpur and subjected to RPA-PCRD strip-based assay for the detection of MoT. (a) photographs of symptomatic weed samples (b) RPA-PCRD strip-based assay for the detection of MoT in weed samples. Test for each test sample was repeated three times.
